# Scale-free dynamics in human neonatal cortex following perinatal hypoxia

**DOI:** 10.1186/1471-2202-14-S1-P36

**Published:** 2013-07-08

**Authors:** James A Roberts, Kartik K Iyer, Simon Finnigan, Sampsa Vanhatalo, Michael Breakspear

**Affiliations:** 1 Systems Neuroscience Group, Queensland Institute of Medical Research, Herston, Brisbane, QLD 4006, Australia; 2 Centre for Clinical Research and Perinatal Research Centre, University of Queensland, Brisbane, QLD 4006, Australia; 3 Department of Children's Clinical Neurophysiology, Helsinki University Central Hospital and University of Helsinki, Helsinki, Finland

## 

Complications at birth can interrupt blood supply to the baby, leading to hypoxia in the neonatal cortex. Once oxygen supply resumes, cortical activity follows a stereotypical recovery sequence that includes a period termed burst suppression, during which the EEG exhibits sudden, irregular fluctuations of highly variable size and shape. Clinical outcome depends critically on this phase, ranging from complete recovery to permanent cognitive or motor disability and even death. Despite its importance in the recovery process, burst suppression's mechanisms remain poorly understood, and objective diagnostics are needed to guide treatment [1].

**Figure 1:**
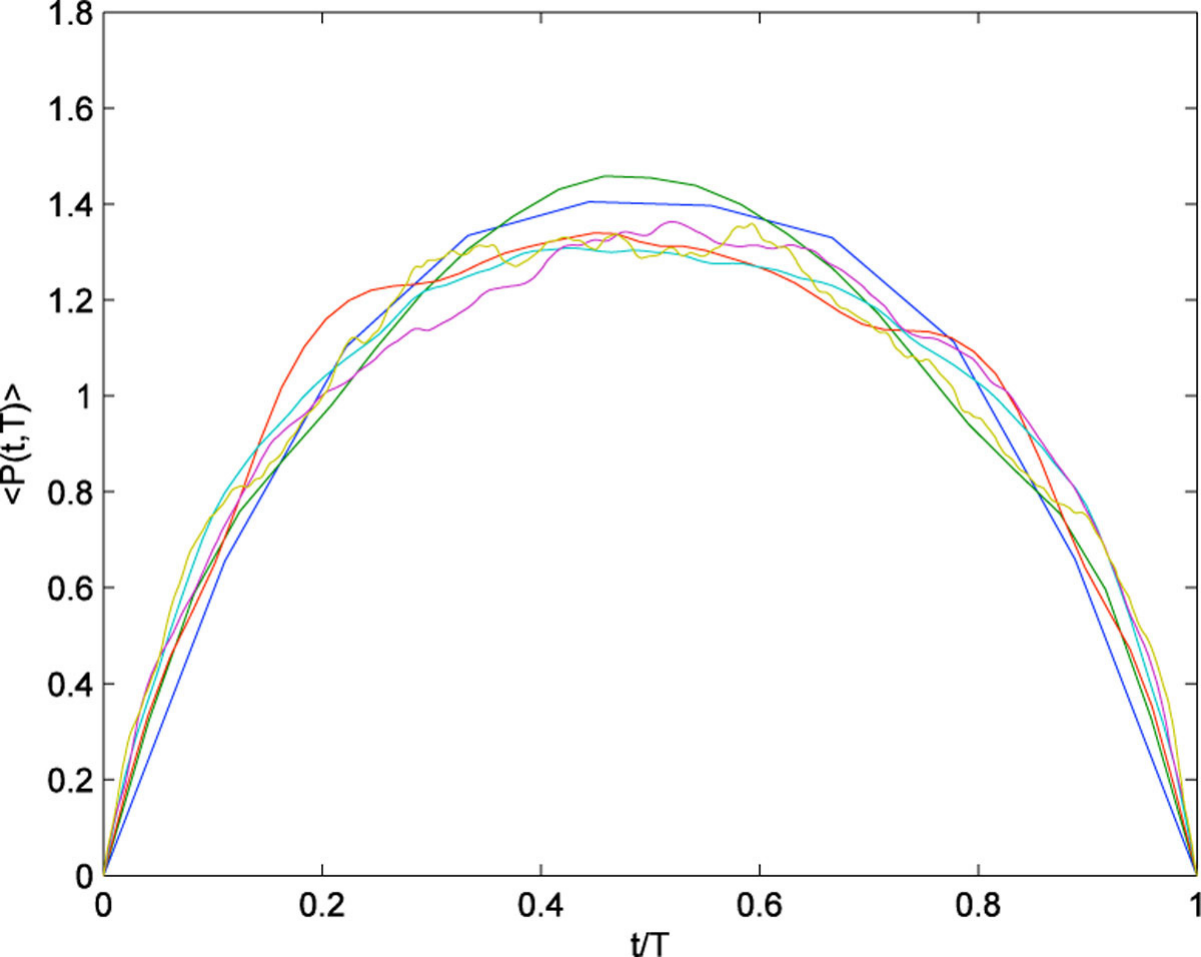
**Example of single-subject average burst shapes collapsing to a simple symmetric functional form over temporal scales of T = 40 ms - 4 s**.

Here, we analyze the statistical properties of burst suppression in neonatal EEG recordings and show that simple dynamical models capture key features of the data. We find that fluctuations in burst size exhibit long-tailed power law distributions spanning up to five orders of magnitude. Despite this immense variability, their average shape at all temporal scales can be rescaled to a near universal template (Figure 1). Deviations from universality include a flattening of fluctuation shapes at long time scales and the expression of leftward or rightward asymmetry. These features are consistent with the phenomenon of crackling noise that arises in disparate physical systems such as crumpling paper, magnetizing a ferromagnet, and earthquakes, all of which exhibit scale-free bursty events [2]. Similar behavior has recently been observed in neuronal avalanches recorded in cortical slices [3]. In our data, as in studies of crackling noise, the average shapes shed light on the underlying mechanisms [4]. Using simple phenomenological models, we show how changes to the average shapes can arise from different forms of state-dependent damping, representing resource depletion in cortical neurons. Statistical analysis of the variability and average shapes of bursts holds promise for new diagnostic opportunities in this critical clinical window and will inform future biologically-detailed models.
